# Which style of duodenojejunostomy is better after resection of distal duodenum

**DOI:** 10.1186/s12893-022-01850-2

**Published:** 2022-11-24

**Authors:** Wenshuai Liu, Jiongyuan Wang, Lijie Ma, Aobo Zhuang, Jing Xu, Junyi He, Hua Yang, Yuan Fang, Weiqi Lu, Yong Zhang, Hanxing Tong

**Affiliations:** 1 Department of General Surgery, Zhongshan Hospital, Fudan University, 200032 Shanghai, People’s Republic of China; 2 Department of General Surgery, South Hospital of the Zhongshan Hospital/Shanghai Public Health Clinical Center, Fudan University, 200083 Shanghai, People’s Republic of China

**Keywords:** Duodenojejunostomy, Duodenum, Retroperitoneal sarcoma, Gastrointestinal stromal tumor

## Abstract

**Background:**

Distal duodenal resections are sometimes necessary for radical surgery, but how to restore duodenal continuity is still unclear. This study aimed at determining which style of anastomosis was more suitable for the duodenojejunostomy after resection of distal duodenum.

**Patients and methods:**

We retrospectively identified 34 patients who underwent distal duodenum resection at our center between January 2014 and December 2021. According to whether the end or the side of the proximal duodenum was involved in reconstruction, duodenojejunostomy were classified as End style (E-style) and Side style (S-style). Demographic data, clinicopathological details, and postoperative complications were analyzed between two groups.

**Results:**

Thirteen patients (38.2%) received E-style duodenojejunostomy, and 21 patients (62.8%) received S-style duodenojejunostomy. Comparative analysis showed that in group of E-style, patients had a lower rate of multivisceral resection(5/13 vs 18/21; *P* = 0.008), delayed gastric emptying (DGE) (1/13 vs 11/21; *P* = 0.011) and intraperitoneal infection (2/13 vs 12/21; *P* = 0.03). In this study, the incidence of major complications was up to 35.3% (12/34) and no patient died of complication in perioperative period. In two group, there was no difference in the incidence of major complications (E-style vs S-style: 3/13 vs 9/21; *P* = 0.292).

**Conclusions:**

The E-style duodenojejunostomy for the reconstruction of distal duodenum resection is safe and feasible. The E-style anastomosis may have potential value in decreasing the occurrence of complications such as DGE and intraperitoneal infection, and the definitive advantages still need to be verified.

## Background

Lesions which originate from or invade distal duodenum, 3rd and 4th segments of duodenum, are commonly tricky and the surgical treatment of them are full of challenges. Addition to primary adenocarcinoma [1] and gastrointestinal stromal tumors (GIST) [2], some diseases invading distal duodenum, such as retroperitoneal sarcoma (RPS) [3] and infections [4], may require duodenectomy and duodenojejunostomy. In the setting of distal duodenum resection, particularly combined with other organs resection, selecting the optimal procedure is extremely important due to the complex anatomical structure of the pancreatic head and duodenum, including superior mesenteric vessels, portal vein, and extrahepatic biliary system. Furthermore, by the same reason, the reconstruction method after segmental resection of the distal duodenum has not yet been standardized. Additionally, the duodenal jejunal anastomosis needs to suffer strong corrosive digestive fluid containing gastric acid, pancreatin and bile acid. If complication, such as anastomotic fistula, surgical site infection or postoperative bleeding occurs in this area, it will lead to catastrophic consequences and even death. Thus, a proper pattern of anastomosis may enhance the postoperative recovery of patients. Herein, we present the short-term results of a case series with the resection of the distal duodenum for various lesions and try to illuminate which style of duodenojejunostomy is a better alternative in restore of the digestive tract.

## Methods

### Patients’ inclusion criteria

**Fig. 1:**
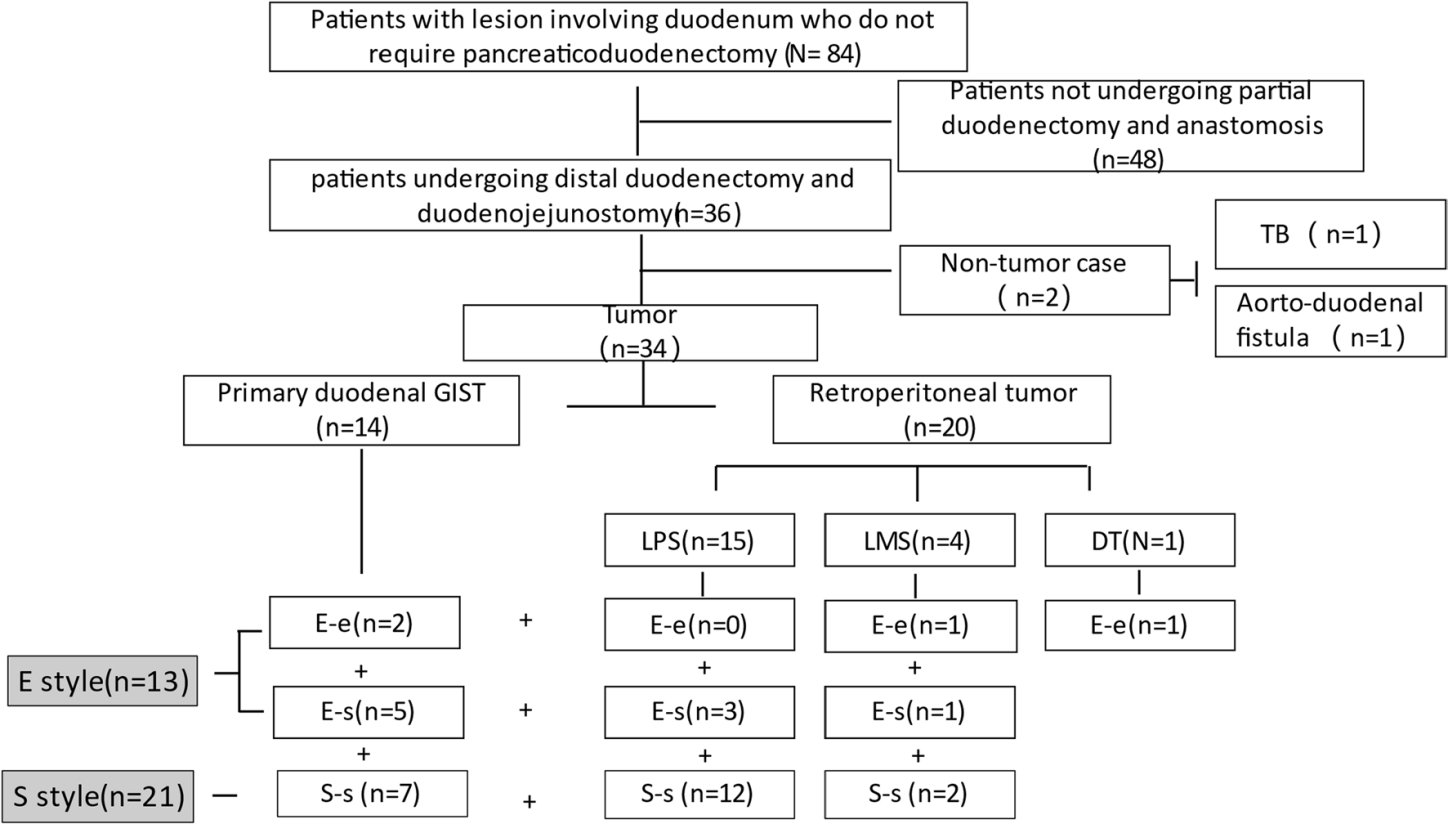
Flowchart of patients enrolled in the study about the style of duodenojejunostomy

We retrospectively reviewed all patients who underwent distal duodenectomy between January 2014 and December 2021 at Retroperitoneal Sarcoma Center of Zhongshan Hospital, Fudan University. This study was designed according to the ethical guidelines of the Helsinki Declaration and was approved by the institutional Ethic Committee. Patients who underwent endoscopic excision, wedge excision, repair, emergency surgery, trans-duodenal ampullectomy, pancreaticoduodenectomy and those with non-neoplastic diseases were all excluded from the study (flowchart for details in Fig. 1).

### Style and procedure of duodenojejunostomy

**Fig. 2:**
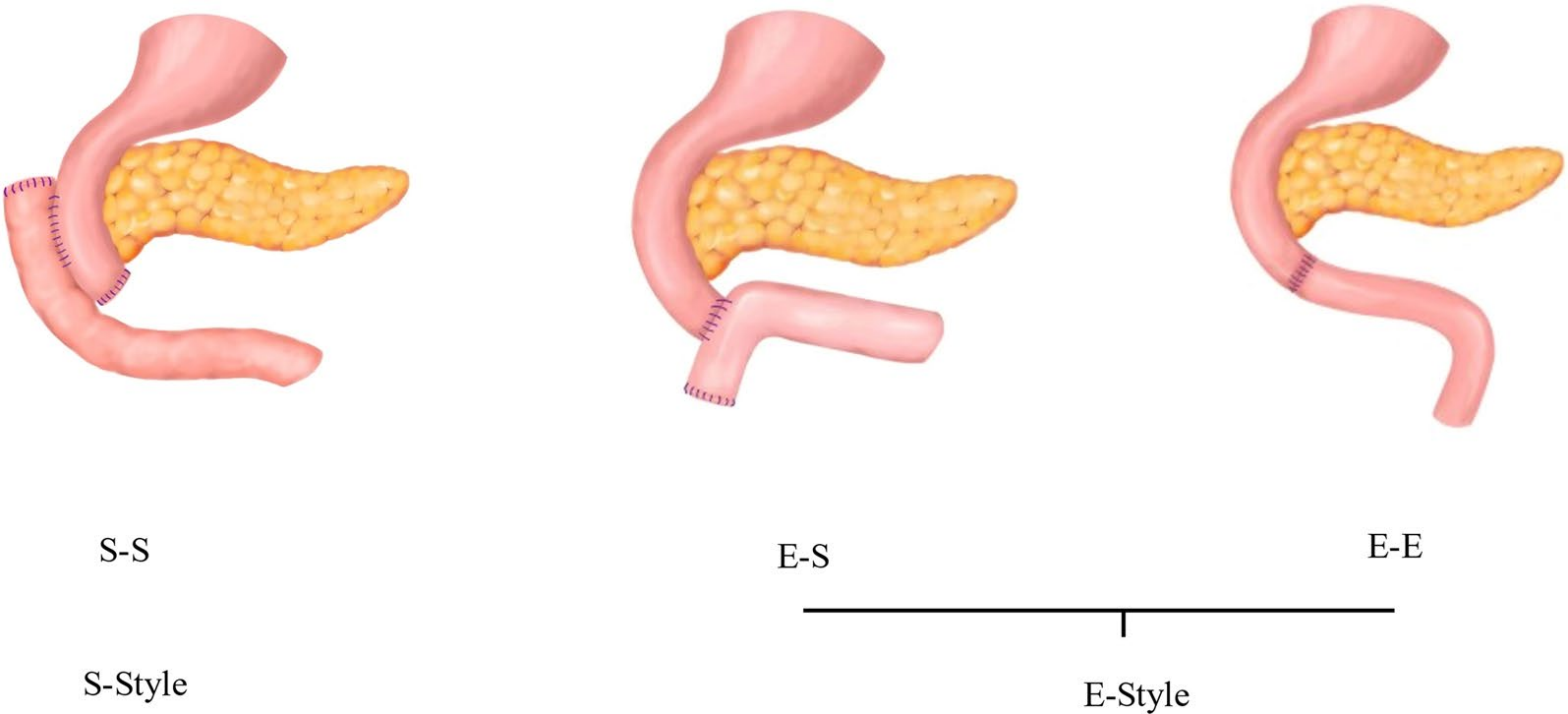
Classification of duodenojejunostomy: S–S, Side-Side anastomosis; E–S, End-Side anastomosis; E–E, End-End anastomosis. According to whether the END or the SIDE of the proximal duodenum was involved in reconstruction, duodenum-jejunum anastomosis was classified as End style (E-style) and Side (S-style)

Different style of duodenojejunostomy was demonstrated in Fig. 2. In brief, side-to-side, (S–S) style duodenojejunostomy was realized as the following procedure: 1. The distal duodenum was closed by a linear stapler; 2. The side-to-side duodenojejunostomy was accomplished via tubular stapler; 3. The jejunal stump was closed via a linear stapler. The procedure of end-to-side (E–S) style duodenojejunostomy was accomplished as follows: 1. The tubular stapler was used to completed anastomosis of the stump of duodenum and the side of proximal jejunum; 2. The proximal jejunal stump was closed by a linear stapler. The end-to-end (E–E) style anastomosis of duodenum and proximal jejunum was realized by hand suture. As illustrations in Fig. 2, according to whether the END or the SIDE of the proximal duodenum was involved in reconstruction, the patterns of duodenum-jejunum anastomosis were classified as End style (E-style) and Side style (S-style).

### Perioperative evaluation

Demographic data, peri-operative and pathological details were collected. Postoperative complications were classified by the Clavien-Dindo classification [5]. And the diagnosis of the pancreatic fistula was according to the International Study Group on Pancreatic Surgery (ISGPS) definition [6].

### Statistical analysis

Statistical analysis was carried out using IBM SPSS Statistics 25. Clinicopathological characteristics were summarized by frequencies and percentages for categorical variables and mean ± standard deviation (SD) for continuous variables. Chi square test was used to compare categorical, whereas Student’s t test and Mann–Whitney U test were used to compare continuous variables. All tests were two-tailed, and result with a P value < 0.05 was considered statistically significant.

## Results

### General characteristics of enrolled patients

According to the above criteria (Fig. 1), 34 patients were finally enrolled in this study, and the features of the 34 patients were shown in Table1. Male patient accounted for 55.9% cases. The mean age was 56.29 ± 12.71 years. The diagnosis were liposarcomas (LPS) in 15 (44.1%), leiomyosarcoma (LMS) in 4 (11.8%) and desmoid tumor (DT) in 1 (2.9%). The above lesions all located in retroperitoneal space and invaded third and fourth segments of duodenum. Others were GIST in 14 (41.2%), which derived from the wall of distal duodenum.

**Table 1:** Clinicopathological characteristics of the enrolled patient

Clinical parameter	
Gender	
Male	19 (55.9%)
Female	15 (44.1%)
Age (mean ± SD, year)	56.29 ± 12.71
Primary or Recurrence	
Primary	18 (52.9%)
Recurrence	16 (47.1%)
Multiple Lesions	
Yes	11 (32.4%)
No	23 (67.6%)
Tumor Size (mean ± SD, cm)	19.0 ± 12.7
Histology	
LPS	15 (44.1%)
GIST	14 (41.2%)
LMS	4 (11.8%)
DT	1 (2.9%)
Multivisceral Resection	
Yes	23 (67.6%)
No	11 (32.4%)
Combined Pancreatic Resection^*^	
Yes	9 (26.5%)
No	25 (73.5%)
Anastomosis Style	
S-style	21 (62.8%)
E-style	13 (38.2%)
Operation Time (mean ± SD, min)	344 ± 121
Intraoperative Blood Loss (mean ± SD, ml)	1294 ± 1342
Complication	
Grade 1–2	22 (64.7%)
Grade 3–5	12 (35.3%)
Delayed Gastric Emptying	
Yes	12 (35.3%)
No	22 (64.7%)
Incision Infection	
Yes	11 (32.4%)
No	23 (67.6%)
Intraperitoneal Infection	
Yes	14 (41.2%)
No	20 (58.8%)
Duodenojejunal anastomotic Fistula	
Yes	6 (17.6%)
No	28 (82.4%)
Pancreatic Fistula	
Yes	7 (20.6%)
No	27 (79.4%)
Postoperative Massive Hemorrhage	
Yes	3 (8.8%)
No	31 (91.2%)
Re-operation	
Yes	3 (8.8%)
No	31 (91.2%)
Time to Feed (mean ± SD, day)	18.8 ± 20.1
Postoperative Hospital stay (mean ± SD, day)	27.0 ± 20.2

LPS: liposarcoma; LMS: leiomyosarcoma; GIST: gastrointestinal stromal tumor; DT: desmoid tumor; SD: stand deviation

*Refers to the removal of the uncinate or the body and tail of the pancreas

High tendency to local recurrence is an important clinical feature of retroperitoneal soft tissue sarcomas. In this study, 16 cases (47%) presented with recurrent tumors, and 11 cases (32.4%) had multiple lesions. In all cases, 23 (67.7%) patients underwent at less two organs resection (multivisceral resection). The operation time (344.12 ± 121.33 min), and intraoperative blood loss (1294.12 ± 1341.65 ml) also reflected that it was very difficult and challenging to remove the tumors in this area.

In this study, almost all patients had postoperative complications of varying grades according to Clavien-Dindo classification [5]. Although majority patients (n = 22, 64.7%) experienced minor postoperative complication (grade 2 or less) and one third patients (n = 12, 35.3%) suffered more serious postoperative complications (grade 3 or greater), there was no patient dead in perioperative period. Intraperitoneal infection (41.2%) was the most common postoperative complication, following by delayed gastric emptying (DGE) (35.3%) and wound complication (32.4%). It’s worth noting that 6 (17.6%) patients developed slight duodenal leakage in the current study, but no patient needed surgical intervention. Nine patients simultaneously underwent partial pancreatectomy, 7 patients suffered grade B or C pancreatic fistula according to International Study Group on Pancreatic Surgery (ISGPS) definition [6] and the patients all recovered via local draining and conservative medical treatment. Three patients developed massively postoperative hemorrhage, and underwent exploratory laparotomy and hemostasis. Patients had a different extent of a long time to feed (18.8 days on average) and postoperative hospital stay (27.0 days on average).

### Comparison of E-style and S-style duodenojejunostomy

In the study, E-style and S-style anastomosis were accounted for 38.2% (13 cases) and 62.8% (21 cases), respectively. To analyze the influence of different duodenum-jejunum anastomosis pattern on postoperative recovery, we further compared the differences in clinicopathological features and postoperative outcomes of two styles of anastomosis (Table 2). Compared with patients with S-style duodenum-jejunum anastomosis, the patients with E-style ananstomosis displayed decreased rate of intraperitoneal infection (5/13 vs 18/21, *P* = 0.030) and less likelihood of DGE (1/13 vs 11/21, *P* = 0.011). It could not be ignored that S-style group had some disadvantages, such as the patients much elder (61.10 vs 50.15, *P* = 0.024) and higher probability of multivisceral resection (18/21 vs 5/13; *P* = 0.008). Although there was no significant statistical difference in the incidence of anastomotic fistula between the two groups, five cases of fistula occurred in the S-style group and only one in E-style group. It should be noted that digestive tract radiography showed one cases of fistula occurred in anastomosis and four in duodenal stump in S-style group.

**Table 2:** Comparison of clinicopathological features and postoperative outcomes between two Duodenojejunostomy styles

	Duodenojejunostomy Styles		*P *value
	S-style	E-style	
Gender			0.728
Male	10 (47.6%)	5 (38.5%)	
Female	11 (52.4%)	8 (61.5%)	
Age (mean ± SD, year)	60.10 ± 11.03	50.15 ± 13.24	0.024
Primary or Recurrence			0.126
Primary	9 (42.9%)	9 (69.2%)	
Recurrence	12 (57.1%)	4 (30.8%)	
Multiple Lesions			0.301
Yes	8 (38.1%)	3 (23.1%)	
No	13 (61.9%)	10 (76.9%)	
Tumor Size (mean ± SD, cm)	20.4 ± 11.8	16.6 ± 14.1	0.397
Histology			0.152
LPS	12 (57.1%)	3 (23.1%)	
GIST	7 (33.3%)	7 (53.8%)	
LMS	2 (9.5%)	2 (15.4%)	
DT	0 (0.0%)	1 (7.7%)	
Multivisceral Resection			0.008
Yes	18 (85.7%)	5 (38.5%)	
No	3 (14.3%)	8 (61.5%)	
Combined Pancreatic Resection*			0.229
Yes	7 (33.3%)	2 (15.4%)	
No	14 (66.7%)	11 (84.6%)	
Operation Time (mean ± SD, min)	358 ± 98	322 ± 154	0.418
Intraoperative Blood Loss (mean ± SD, ml)	1552 ± 1292	877 ± 1364	0.165
Complication			0.292
Yes	12 (57.1%)	10 (76.9%)	
No	9 (42.9%)	3 (23.1%)	
Delayed Gastric Emptying			0.011
Yes	11 (52.4%)	1 (7.7%)	
No	10 (47.6%)	12 (92.3%)	
Incision Infection			0.465
Yes	8 (38.1%)	3 (23.1%)	
No	13 (61.9%)	10 (76.9%)	
Intraperitoneal Infection			0.030
Yes	12 (57.1%)	2 (15.4%)	
No	9 (42.9%)	11 (84.6%)	
Duodenojejunal anastomotic Fistula			0.370
Yes	5 (23.8%)	1 (7.7%)	
No	16 (76.2%)	12 (92.3%)	
Pancreatic Fistula			0.210
Yes	6 (28.6%)	1 (7.7%)	
No	15 (71.4%)	12 (92.3%)	
Postoperative Massive Hemorrhage			1.000
Yes	2 (10.5%)	1 (7.7%)	
No	19 (89.5%)	12 (92.3%)	
Unplanned re-operation			1.000
Yes	2 (10.5%)	1 (7.7%)	
No	19 (89.5%)	12 (92.3%)	
Time to Feed (mean ± SD, day)	22.0 ± 16.0	13.8 ± 25.4	0.255
Postoperative Hospital Stay (mean ± SD, day)	30.5 ± 14.8	21.4 ± 26.6	0.208

LPS: liposarcoma; LMS: leiomyosarcoma; GIST: gastrointestinal stromal tumor; DT: desmoid tumor; SD: stand deviation

*Refers to the removal of the uncinate or the body and tail of the pancreas

There is no statistical difference in the recurrence rate (57.1% vs 30.8%), multiple lesion (38.2% vs 23.1%, *P* = 0.301), tumor size (20.4 cm vs 16.4 cm, *P* = 0.397) and histological distribution (*P* = 0.152) between E-style and S-style duodenojejunostomy group. Compared to the E-style group, there is no significance in the major complication (42.9% vs 23.1%, *P* = 0.292), incision infection (38.1% vs 23.1%, *P* = 0.465), duodenojejunal anastomotic fistula (23.8% vs 7.7%, *P* = 0.370), pancreatic fistula (28.7% vs 7.7%, *P* = 0.210), postoperative massive hemorrhage (10.5% vs 7.7%, *P* = 1.000) and consequent unplanned reoperation(10.5% vs 7.7%, *P* = 1.000) in the postoperative recovery phase. The E-style duodenojejunostomy group had a shorter time to feed (13.8 days vs 22.0 days, *P* = 0.255) and postoperative hospital stay (21.4 days vs 30.5 days, *P* = 0.208), although no statistically significant was reached. The operation time (358 min vs 322 min, *P* = 0.418) and volume of blood loss (1552 ml vs 877 ml, *P* = 0.165) in both anastomosis style groups were similar, no significant difference were observed.

### Major complication analysis

As Table 1 showed, in this study, the incidence of complications was relatively high, and the incidence of major complications was as high as 35.3%. In Table 3, we further analyzed the factors which could affecting the occurrence of major complication and the results indicated that recurrence disease (15/22 vs 3/12, *P* = 0.030), multiple lesions (19/22 vs 4/12, *P* = 0.005), tumor size (28.99 vs 13.48, *P* $<$ 0.001), histology (10/15 vs 2/14 vs 0/4 0/1, *P* = 0.005), multivisceral resection (12/12 vs 11/22, *P* $<$ 0.03), combined pancreatic resection (6/12 vs 3/22, *P* = 0.040), longer operative time (432.17 vs 296.09, *P* $=$ 0.008) and high volume of intraoperative blood loss (2480.83 vs 646.82, *P* $=$ 0.001) were all associated with the occurrence of severe postoperative complication (Grade 3–5) during recovery. The major complication had no relationship to gender (*P* = 0.288), age (*P* = 0.481) and duodenojejunostomy style (*P* = 0.292). But Multivariate analysis showed that there were no independent influencing factors for the recurrence of major complications. Further analysis indicated that For patients with LPS, the incidence of major complications being up to 66.67%, were far higher than others (10/15 vs 2/19, *P* = 0.001), the proportion of multivisceral resection (13/15 vs 10/19; *P* = 0.039) and S-style anastomosis (12/15 vs 9/19; *P* = 0.055) were higher than in others, but styles of anastomosis did not influenced occurrence of major complications(S-style vs E-style, 8/12 vs 2/3, Fish exact = 1.000).

**Table 3:** Univariate analysis of factors affecting the occurrence of major complications

	Complication		*P value*
	Grade 1–2	Grade 3–5	
Gender			
Male	8 (53.3%)	7 (46.7%)	0.288
Female	14 (73.7%)	5 (26.3%)	
Age (mean ± SD, year)	55.1 ± 13.6	58.4 ± 11.1	0.481
Primary or Recurrence			0.030
Primary	15 (83.3%) 7 (43.8%)	3 (16.7%) 9 (56.2%)	
Recurrence			
Multiple Lesion			0.005
Yes	19 (82.6%) 3 (27.3%)	4 (17.4%) 8 (72.7%)	
No			
Size (mean ± SD, cm)	13.5 ± 9.6	29.0 ± 11.6	< 0.001
Histology			0.005
LPS	5 (33.3%) 12 (85.7%)	10 (66.7%) 2 (14.3%)	
GIST			
LMS	4 (100.0%)	0 (0.0%)	
DT	1 (100.0%)	0 (0.0%)	
Anastomosis Styles			0.292
S-style	12 (57.1%) 10 (76.9%)	9 (42.9%) 3 (23.1%)	
E-style			
Multivisceral Resection			0.030
Yes	11 (47.8%) 11 (100.0%)	12 (52.2%) 0 (0.0%)	
No			
Combined Pancreatic Resection			0.040
Yes	3 (33.3%) 19 (76.0%)	6 (66.7%) 6 (24.0%)	
No			
Operation Time (mean ± SD, min)	296 ± 75	432 ± 143	0.008
Intraoperative Blood Loss (mean ± SD, ml)	647 ± 799	2481 ± 1344	0.001

## Discussion

In the May 1922, duodenojejunostomy was originally designed to drain the duodenal contents into the small intestines whenever some obstacle hindered the evacuation of the duodenal contents. Subsequently, in order to avoid enteric recycling caused by omega loop and promote duodenal emptying, the original side-to-side anastomosis was replaced by the modified side-to-end anastomosis [7]. With the development of modern surgery, the resection of lesions originating from the region of the distal duodenum, such as duodenal adenocarcinoma [1], GISTs [2], RPS [3], and even abdominal aortoduodenal fistula [4], etc. was no longer impossible. Due to the complexity of the anatomy, the operation was difficult, and usually required the removal of lesions companied with the distal portion of the duodenum, even more organs. Followed removal of lesions, restoring continuity of the duodenum became imperative. By reviewing our data and referring to relevant literatures [8–11], we classified duodenum-jejunal anastomosis as three procedures, (Fig. 2) but which one was more advantageous was still controversial. The side-to-side anastomosis, resulting in blind pouch syndrome, has long been noted [12]. This term is used to describe the long-term complications of side-to-side procedure. It leads to the progressive distention of the cul-de-sac, which produces definite pouch of stasis and bacterial infection [13]. In principle, the reconstruction of the gastrointestinal tract should be performed by end-to-end anastomosis whenever possible, and a blind pouch should be avoided as much as possible to prevent the development of the blind pouch syndrome [8].

End-to-end and end-to-side procedures for duodenojejunostomy could avoid the development of blind pouch, so they were classified into the same class and named E-style anastomosis in this study. Meanwhile, the anastomosis used side-side technique was named S-style. (Fig. 2). Which style anastomosis is more advantageous in decreasing occurrence of postoperative complications? This study retrospectively analyzed 34 cases of tumors located in the area of the distal duodenum in our center, and compared the postoperative recovery of two different anastomosis styles. The results showed that the incidence of postoperative DGE and intraperitoneal infection was significantly decreased in E-style group, but the rate of multivisceral resection was much higher in S-style group. In addition, in the S-style group, four cases of duodenal stump fistula and one case anastomotic fistula occurred, as for the E-style group, only one case of duodenal fistula occurred. According to reference 13, we speculated that the presence of the blind pouch would weaken the emptying capacity of the duodenum, and result in increased tension and intestinal dilation at the anastomotic site, and which will increase the risk of anastomotic fistula in S-style anastomosis. In practice, we often do not dare to use the stump of the duodenum to perform E-style anastomosis, due to the presence of vascular gap in distal duodenum and combined pancreatic resection [7]. Moreover, Due to popularity of staplers, it has been became much easier to performed anastomosis on the fully motived descending segment of duodenum. Thus, this study reminds us to ponder our perspective on duodenojejunostomy, and avoidance of S-style anastomosis may be an optimal option.

RPS is a kind of refractory tumor with various pathological classification and has high tendency of local relapse [14, 15]. GISTs are the most common mesenchymal tumors of the digestive tract and represent 1–3% of all digestive tract neoplasms, but which located in distal duodenum is rare [16, 17]. For both RPS and GIST, surgical excision is the cornerstone of treatment. In this study, RPS and GISTs were all located in the retroperitoneal space and peri-duodenal region, and were required resection companied with the distal duodenum. Usually, Kocher and Cattell-Braash maneuvers were used to fully expose retroperitoneal space [18]. Followed by mobilization of the ligament of Treitz and retro-rotation of the root of mesentry, tumor with the horizontal portion of the duodenum could be evaluated to en-bloc resect. It is crucial to determine the location of the duodenal papilla before excision and anastomosis to prevent accidental injury. Additionally, invasion of adjacent organ will result in multivisceral resection, including pancreas, colon, small bowel, and kidney etc. For RPS resection, a complicated surgical procedure, the incidence of postoperative complications is high [19]. In our study, the incidence of major complications was as high as 35.3%. For patients with LPS, the rate of major complications being up to 66.67% (10/15), were far higher than others. Univariate analysis showed that recurrence tumor, multiple lesions, tumor size, combined multivisceral resection, pancreatic resection, operation time, and intraoperative blood loss all affected the occurrence of major complication.

Our study suggested that tumor burden and surgical trauma may have an important role in the occurrence of major complications. Statistically, the anastomosis style does not affect the occurrence of postoperative major complications, but E-style anastomosis may have potential value in decreasing the occurrence of complications such as DGE and intraperitoneal infection.

The resection of peri-distal duodenum tumor is complicated and the relevant studies are few. Cananzi et al. [3] analyzed technical aspects and post-operative outcomes in patients with RPS and GIST involving duodenum. Thirty patients were treated: 15 for GIST, 15 for RPS. Sixteen duodenal wedge resections and 14 segmental resections were performed. Multivisceral resection was frequently performed (63.4%). Overall postoperative complication rate was 53% (16/30) and duodenum-related complication rate was 33% (10/30). It was obvious that duodenal resections for RPS and GIST have significant morbidity rate. Regretfully, the article did not analyze the pattern of duodenojejunostomy.

The purpose of this study is to determine which style of anastomosis is more advantageous after resection of distal duodenum in the setting of surgery for retroperitoneal tumors. Due to the demerits of this study, such as retrospective study, small size of samples and imbalanced influencing factors, the potential advantages of E-type anastomosis need to be further validated. We hope that this study will inspire research on this clinical issue.

## Conclusion

Theoretically, the E-style duodenojejunostomy avoids a blind pouch and prevents the development of blind pouch syndrome. The E-style anastomosis may have potential value in decreasing the occurrence of complications such as DGE and intraperitoneal infection, and the definitive advantages still need to be verified.

## Data Availability

All data generated or analysed during this study are included in its additional files.
